# Junín Virus Infection Activates the Type I Interferon Pathway in a RIG-I-Dependent Manner

**DOI:** 10.1371/journal.pntd.0001659

**Published:** 2012-05-22

**Authors:** Cheng Huang, Olga A. Kolokoltsova, Nadezdha E. Yun, Alexey V. Seregin, Allison L. Poussard, Aida G. Walker, Allan R. Brasier, Yingxin Zhao, Bing Tian, Juan Carlos de la Torre, Slobodan Paessler

**Affiliations:** 1 Galveston National Laboratory, Department of Pathology and Institute for Human Infections and Immunity, University of Texas Medical Branch, Galveston, Texas, United States of America; 2 Department of Internal Medicine and Sealy Center for Molecular Medicine, Institute for Translational Sciences, University of Texas Medical Branch, Galveston, Texas, United States of America; 3 Department of Internal Medicine, University of Texas Medical Branch, Galveston, Texas, United States of America; 4 Department of Immunology and Microbial Science, The Scripps Research Institute, La Jolla, California, United States of America; University of Texas Medical Branch, United States of America

## Abstract

Junín virus (JUNV), an arenavirus, is the causative agent of Argentine hemorrhagic fever, an infectious human disease with 15–30% case fatality. The pathogenesis of AHF is still not well understood. Elevated levels of interferon and cytokines are reported in AHF patients, which might be correlated to the severity of the disease. However the innate immune response to JUNV infection has not been well evaluated. Previous studies have suggested that the virulent strain of JUNV does not induce IFN in human macrophages and monocytes, whereas the attenuated strain of JUNV was found to induce IFN response in murine macrophages via the TLR-2 signaling pathway. In this study, we investigated the interaction between JUNV and IFN pathway in human epithelial cells highly permissive to JUNV infection. We have determined the expression pattern of interferon-stimulated genes (ISGs) and IFN-β at both mRNA and protein levels during JUNV infection. Our results clearly indicate that JUNV infection activates the type I IFN response. STAT1 phosphorylation, a downstream marker of activation of IFN signaling pathway, was readily detected in JUNV infected IFN-competent cells. Our studies also demonstrated for the first time that RIG-I was required for IFN production during JUNV infection. IFN activation was detected during infection by either the virulent or attenuated vaccine strain of JUNV. Curiously, both virus strains were relatively insensitive to human IFN treatment. Our studies collectively indicated that JUNV infection could induce host type I IFN response and provided new insights into the interaction between JUNV and host innate immune system, which might be important in future studies on vaccine development and antiviral treatment.

## Author Summary

Junín virus (JUNV), which is endemic to the Argentinean Pampas region, is the causative agent of Argentine hemorrhagic fever (AHF), a severe illness with hemorrhagic and neurological manifestations and with a case fatality of 15–30%. Clinical studies demonstrate that elevated levels of interferon and cytokines are produced in AHF patients, which might be correlated to the severity of disease. However it remains unclear, especially during virus infection, how human cells can sense virus infection and respond by activation of IFN pathway. Our studies clearly demonstrated that JUNV infection could activate type I IFN response in human cells. IFN pathway activation occurred in cells infected with either virulent strain or attenuated vaccine strain of JUNV. Our data also revealed for the first time that RIG-I was required for type I IFN production during virus infection in human cells. Interestingly, both strains of JUNV were relatively insensitive to human IFN treatment, which might have implications for the role of the IFN on virus infection *in vivo*. Overall, these results indicate that JUNV infection could induce host IFN response and provide new insights into JUNV and host interaction as well as the mechanism underlying AHF.

## Introduction

Arenaviruses are enveloped viruses with a bi-segmented negative strand RNA genome [1]. Each genomic RNA segment, the L (ca. 7.3 kb) and S (ca. 3.5 kb) segments, uses an ambisense coding strategy to direct the synthesis of two proteins in opposite orientations from two open reading frames, which are separated by a non-coding intergenic region (IGR) that acts as a transcription termination signal for the virus polymerase [2], [3]. The viral S segment RNA encodes the viral glycoprotein precursor (GPC) and the nucleoprotein (NP). GPC is post-translationally cleaved by the cellular site 1 protease (S1P) to yield two glycoproteins, GP1 and GP2, which form the viral spikes in the mature virion crucial for receptor recognition and virus entry [4]–[6]. The L segment RNA encodes the viral RNA dependent RNA polymerase (RdRp, or L polymerase) [7], and the small (ca. 11 kDa) RING finger protein Z, the latter representing arenavirus counterpart of the matrix protein found in many other negative strand RNA viruses [8]–[10].

The Arenaviridae family includes several important human pathogens [1], [11]. Viruses often chronically infect their natural rodent hosts worldwide [1]. Infection in humans occurs usually through mucosal exposure to aerosols or by direct contact of abraded skin with infectious materials and may result in severe diseases such as hemorrhagic fever (HF). The Old World (OW) Lassa virus (LASV) and several New World (NW) arenaviruses, including Junín virus (JUNV), pose a serious public health problem within their endemic regions [1], [11]–[13]. JUNV, an agent handled mandatorily in a high-containment biosafety level 4 facility, causes Argentine hemorrhagic fever (AHF), a highly infectious human disease with 15–30% case fatality [13]–[16]. Despite extensive studies in the past, the pathogenesis of HF arenaviruses remains largely uncharacterized.

Viral infections often lead to activation of innate immune response that enables the host to effectively fight against the intruding pathogen [17], [18]. Some of the potent host antiviral responses depend on the production of interferons (IFNs), including type I IFN (IFN-α and IFN-β) and type II IFN (IFN-γ) [19]. Induction of type I IFN is a critical event in the establishment of the antiviral immune response, which results in the reduction of viral spread and subsequent mobilization of adaptive immune responses. During RNA virus infection, IFN response in sentinel cells is initiated by pattern recognition receptors (PRRs) that preferentially recognize viral RNAs typically containing double-stranded RNA structures or 5′ triphosphate termini. Members of the PRR family include cell transmembrane Toll-like receptors (TLRs) (TLR3, TLR7, TLR9), and cytoplasmic RIG-I-like receptors (RLH), RIG-1 and MDA5 [20]–[24]. The ubiquitous RIG-I and MDA5 are the primary PRRs that induce IFN production by activation of Interferon Regulatory Factor 3 (IRF3) via the downstream Mitochondrial Antiviral Signaling protein (MAVS, a.k.a IPS-1/VISA/Cardiff) [22], [23]. Subsequently, newly synthesized IFNs signal through IFN receptor/JAK/STAT pathway to induce the expression of a spectrum of IFN-stimulated genes (ISG). Ultimately, different ISGs execute their antiviral activities in a collaborative manner by targeting various steps of virus replication [25]. Because of the potent antiviral activities mediated by type I IFN, many viruses have evolved strategies to target the RLH-MAVS pathway to evade the host antiviral response [17], [19].

High levels of IFN-α (2000 U/ml–64,000 U/ml) and other cytokines are frequently measured in serum of AHF patients [14], [26]–[30]. High levels of IFN also co-exist with high viremia and severe/fatal diseases [14], [27]. One study has reported that a virulent strain of JUNV does not induce detectable IFN production in human macrophages [31], whereas another study has shown that an attenuated JUNV can activate IFN response in murine macrophages via TLR2 recognition of viral GP protein [32]. The NP protein of JUNV, similar to NPs derived from many other arenaviruses, has been found to exert an anti-IFN activity in cell-based reporter gene assays [33]–[35]. However, the interplay between JUNV and host IFN pathway in the context of virus-infected cells has not been well investigated. Likewise, it is unknown whether pathogenic and non-pathogenic strains of JUNV induce different host innate immune responses. In the present study, we have examined the interaction between JUNV and type I IFN pathway in JUNV-infected human cells. We provide evidence that non-monocyte/macrophage derived human cells efficiently recognized JUNV during the early stages of infection, which resulted in a potent RIG-I-dependent type I IFN response that nonetheless had a very limited effect on JUNV multiplication.

## Materials and Methods

### Viruses

The Romero and Candid#1 strains of JUNV were obtained from Drs. Thomas G. Ksiazek (Centers for Disease Control and Prevention, Atlanta, GA) and Robert Tesh (The World Reference Center for Emerging Viruses and Arboviruses (WRCEVA), University of Texas Medical Branch, Galveston, TX), respectively. Virus stocks were propagated on Vero cells (American Tissue Culture Collection, Manassas, VA), followed by filtration through filters (0.45 µm pore size) to remove cell debris and purification with Ultra 100K Filters Devices (Ultralcel 100K, molecular weight cutoff 100,000, Amicon, Millipore) to remove cellular factors that might mediate innate immune response. All work with the pathogenic Romero strain JUNV was performed in the University of Texas Medical Branch BSL 4 facilities (Robert E. Shope Laboratory or the Galveston National Laboratory) in accordance with institutional health and safety guidelines and federal regulations as described previously [36], [37].

### Quantitative real-time RT-PCR (qRT-PCR)

cDNA synthesis and PCR amplification were performed with iScript cDNA Synthesis Kit and iQ SYBR Green Supermix (Bio-Rad, CA) according to the manufacturer's instructions. Real-time PCR was performed on CFX96 Real-Time PCR Detection System (Bio-Rad, CA). C_t_ values were normalized to the average C_t_ values of glyceraldehyde-3-phosphate dehydrogenase (GAPDH) and actin β (ACTB) housekeeping genes. Primers were designed from target mRNA sequences obtained through NetAffx Analysis Center (Affymetrix, Santa Clara, CA) using NCBI/Primer-BLAST. The sequences of the primers used in qRT-PCR will be available upon request. ΔΔC_t_ based fold-change calculations and statistical analysis (Student's t-test) of quantitative real-time RT-PCR data were performed at the RT^2^ Profiler™ PCR Array Data Analysis Web Portal (http://www.sabiosciences.com/pcr/arrayanalysis.php).

### Quantification of human IFN-β protein in cell culture supernatants

Concentration of human IFN-β was measured by sandwich ELISA method using VeriKine™ Human IFN-Beta ELISA Kit (PBL Interferon Source, NJ) according to the manufacturer's instruction.

### Preparation of cytoplasmic (CE) and nuclear extracts (NE)

A549 cells were scraped and subjected to hypotonic buffer/detergent lysis [38]. The supernatant (CE) was saved and the nuclear extract (NE) was purified by centrifugation through a sucrose cushion followed by extraction in Buffer C (50 mM HEPES, pH 7.9, 10% glycerol, 400 mM KCl, 1 mM EDTA, 1 mM EGTA, 1 mM DTT and 0.1 mM PMSF) with protease inhibitor cocktail (Sigma Aldrich, St. Louis, MO). Protein concentration was estimated by Coomassie Brilliant Blue staining using BSA as a standard (Bio-Rad, Hercules, CA). Samples were further subjected to quantitative proteomic analysis as described in Text S1 and Table S1.

### Virus sensitivity to IFN treatment

Vero cells were seeded into 96-well plates for 24 h and treated with various concentrations of IFN-α-2b (Intron A, Schering Corporation, NJ), IFN-β1a (Sigma-Aldrich, MO) (125, 250, 500, 1000 and 2000 U/ml) or IFN-γ (Sigma-Aldrich, MO) (125, 250, 500 and 1000 U/ml) for 8 h. Cells were infected with Candid#1 or Romero strains of viruses at a multiplication of infection (MOI) of 0.1 PFU/cell or mock-infected. Supernatants were collected at 2 days post infection (p.i.) and virus titers were determined by plaque assay on Vero cells. MTT-based viability assay was performed to ensure that the reduction of virus titer was associated with the antiviral effect but not with the potential cytotoxicity of IFNs. Due to the severe cytotoxicity caused by IFN-γ treatment at 2000 U/ml, virus infection was not performed at this condition.

### Knockdown of gene expression via siRNA

ON-TARGET plus SMART pool siRNA targeting human DDX58 (RIG-I), IRF3 or Non-targeting Pool (Thermo Fisher Scientific Inc, Pittsburgh, PA) were transfected into A549 cells by electroporation using Amaxa Cell Line Nucleofector Kit T (Lonza Walkersville, Inc., MD) according to the manufacturer's protocol. At 24 h post transfection (p.t.), cells were seeded into 12-well plates. At 48 h p.t. cells were mock-infected or infected with Candid#1 virus at an MOI of 1 PFU/cell and incubated for another 24 h. Total RNA was extracted as described earlier. Protein lysate was prepared in parallel in 1×SDS-PAGE loading buffer and subjected to Western blotting analysis.

### Western blotting (WB) and antibodies

Protein samples were resolved on 4–20% SDS-PAGE gel and transferred to PVDF membranes using Mini Trans-Blot Electrophoretic Transfer Cell apparatus (Bio-Rad, CA). The membranes were incubated with primary antibodies overnight at 4°C and then with appropriate secondary antibodies for 1 h at room temperature. Proteins were visualized with ECL Western Blotting Detection Reagents (GE, NJ) according to the manufacturer's instruction. Primary antibodies used for western blotting analysis were rabbit anti-phosphorylated STAT1 antibody (#9171, Cell Signaling), mouse anti-STAT1 antibody (WH0006772M1, Sigma), rabbit polyclonal anti-RIG-I antibody (AP1900a, Abgent Inc., San Diego, CA), rabbit anti-IRF 3 antibody (ab76409, Abcam), and goat anti-human β actin antibody (sc-1616, Santa Cruz Biotechnology). Secondary antibodies used were HRP-conjugated goat anti-rabbit IgG (#7074, Cell Signaling), HRP-conjugated Goat anti-mouse IgG (115-035-146, Jackson Immunology) and HRP-conjugated donkey anti-goat IgG (sc-2020, Santa Cruz).

### IFN reporter assay

Vero cells grown on 12-well plates were co-transfected with IFN reporter plasmid pISRE-luc and renillar luciferase expression vector pRL-SV40 with Lipofectamine 2000 (Invitrogen). At 24 h p.t., cells were mock-infected or infected with Candid#1 JUNV at an MOI of 1 for 24 h. Cells were treated with IFN-α (1000 IU/ml) for 6 h. Cell lysates were prepared and subjected to dual luciferase reporter assay (Dual-Luciferase reporter assay system, Promega) according to the manufacturer's protocol. The ISRE-driven firefly luciferase expression levels were normalized to those of renillar luciferase. All experiments were performed in triplicates.

### Venezuelan equine encephalitis virus (VEEV) TC83-GFP super-infection assay

A549 or Vero cells were infected or mock-infected by Candid#1 JUNV (MOI of 1.0) for 24 h. Then cells were super-infected with VEEV TC83-GFP virus at an MOI of 0.1. At different time points p.i., media from TC83-GFP virus infected cells were harvested and subjected to plaque assay to determine TC83-GFP virus titers in BHK cells. TC83-GFP virus infection resulted in plaque formation in BHK cells at 48 h p.i. For JUNV infection, plaque formation required virus infection for 7 to 8 days in Vero cells. To rule out a possibility that JUNV alone could also form plaques in BHK cells at 48 h p.i, we performed plaque assay in parallel using media collected from cells infected with Candid#1 JUNV alone in super-infection experiments. It was confirmed that no plaque was formed in BHK cells at 48 h when supernatants from cells infected by Candid#1 JUNV alone were tested. As indicated, TC-83 GFP virus-driven GFP expression in infected cells was also assessed by fluorescence microscopy at 24 h p.i.

## Results

### Effect of infection with the non-pathogenic Candid#1 strain of JUNV on activation of the type I IFN pathway

**Figure 1:**
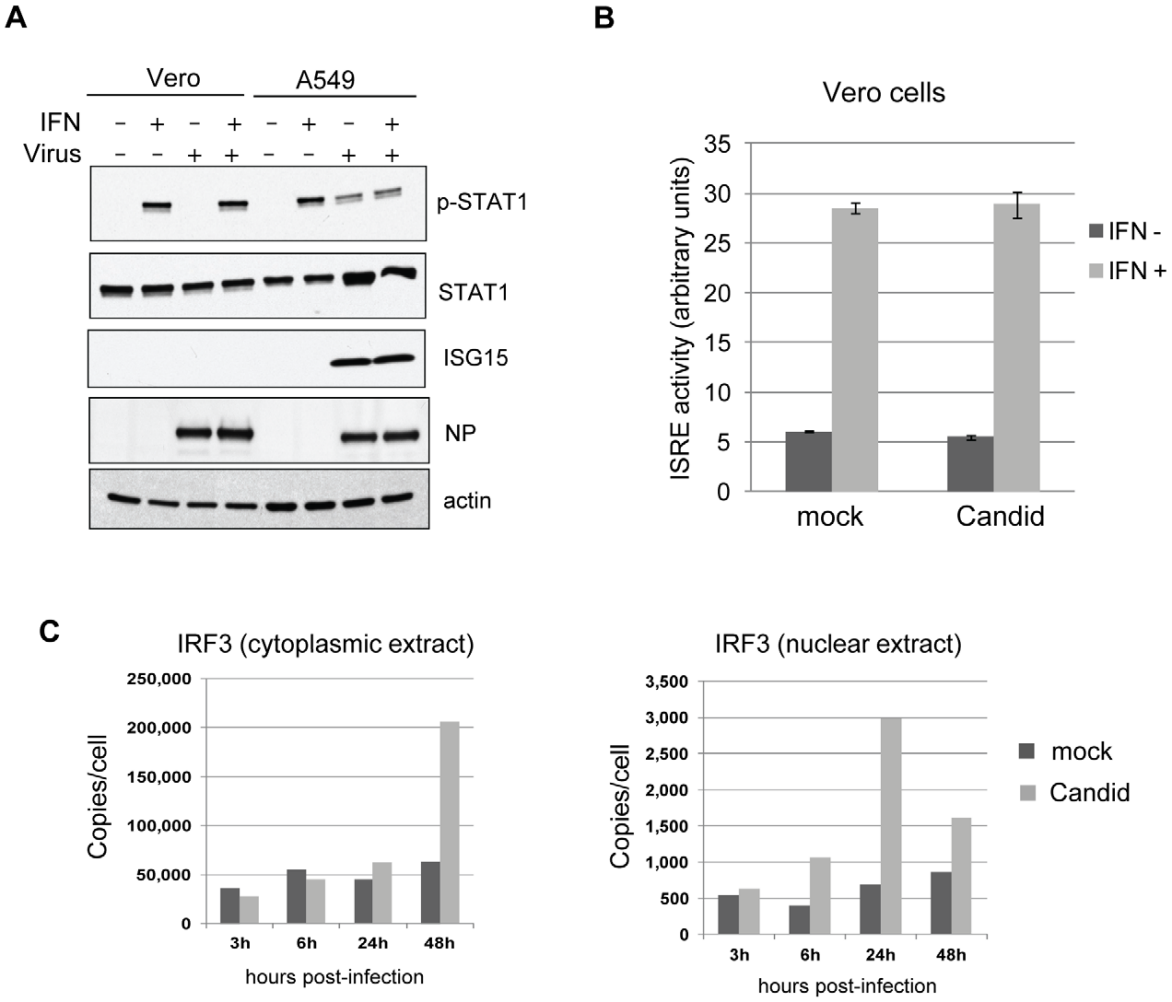
Activation of IFN pathway by Candid#1 JUNV infection. (A) Vero and A549 cells were mock-infected or infected (MOI of 1) with Candid#1 JUNV for 24 h and then mock-treated or treated with IFN-α (1000 IU/ml) for 1 h. Cell lysates were prepared and subjected to western blotting analysis for phosphorylated STAT1 (p-STAT1), STAT1 protein (STAT1), ISG15 (ISG15), virus NP protein (NP) and human β-actin (actin). (B) Vero cells were co-transfected with an IFN responsive IRES reporter plasmid pISRE-luc (expressing firefly luciferase) together with a plasmid constitutively expressing the Renilla luciferase (RL) reporter gene under the Simian Virus 40 promoter (pRL-SV40). After 24 h of transfection, cells were infected with Candid#1 JUNV (Candid) or mock infected (mock) for 24 h followed by mock- (IFN−) or IFN-α treatment (1000 IU/ml) (IFN+) for 6 hr. Cells lysates were prepared and subjected to dual luciferase assay. The ISRE-driven firefly luciferase activity was normalized to the RL activity. Data shown are the mean of three independent experiments (error bar, S.D.). (C) A549 cells were mock infected (mock) or infected with Candid#1 JUNV (Candid). At various time points as indicated, cytoplasmic extract and nuclear extract were prepared for measurement of IRF3 protein level in cytoplasm and nuclear fractions by SID-SRM analysis.

To investigate the interaction between JUNV infection and the type I IFN pathway, we used a human lung epithelial cell line (A549) that has been used extensively in studies of arenavirus infections [34], [35] and likely recreates the situation associated with aerosol transmission of JUNV [1], [16]. In our initial experiments, we used the vaccine strain Candid#1 JUNV, which can be studied in BSL2 laboratories. A549 (IFN-competent) and Vero (IFN-deficient) cells were infected with Candid#1 JUNV (MOI = 1) and analyzed for the expression of ISGs at 24 h p.i.. Candid#1 JUNV infection induced ISG15 protein expression in A549 but not in Vero cells (Figure 1A). The level of STAT1 protein, an ISG, increased noticeably in infected A549 cells but not in Vero cells. Interestingly, phosphorylation of STAT1 protein, a hallmark event of IFN signaling, was evident in Candid#1 JUNV-infected A549, but not in Vero cells (Figure 1A), suggesting that the downstream IFN signaling pathway was activated and responsible for STAT1 phosphorylation.

To investigate the effect of virus infection on type I IFN signaling, Vero cells were infected with Candid#1 JUNV for 24 h and then treated with 1000 u/ml of IFN-α for 1 h. IFN treatment led to STAT1 phosphorylation that was not affected by infection with Candid#1 strain of JUNV (Figure 1A) in Vero cells. At this time point, ISG15 protein expression was still too early to be detected as expected. Similarly, Candid#1 JUNV infection did not affect IFN-α induced expression of an ISRE-dependent reporter gene (pISRE-luc) in transfected Vero cells (Figure 1B). These findings indicated that Candid#1 JUNV infection has no detectable impact on type I IFN signaling.

Early activation of the IFN-β promoter involves the activation and nuclear translocation of IRF3, which is critical for type I IFN response. Our finding that the IFN pathway was activated during Candid#1 JUNV infection led us to further investigate nuclear translocation of the endogenous IRF3 in infected cells. Our initial studies using immunofluorescence microscopy did not show significant levels of IRF3 translocation into nucleus in Candid#1 JUNV-infected cells, whereas IRF3 nuclear translocation was readily detected in cells transfected with poly (IC) (data not shown). We reasoned that this might reflect a low level of IRF3 activation during virus infection, which might be difficult to discern by immunofluorescence staining. Therefore, we examined IRF3 protein levels and subcellular distribution in Candid#1 JUNV-infected cells by a highly specific stable isotopic dilution (SID)-selected reaction monitoring (SRM)-mass spectrometry analysis [38]–[40]. Cytoplasmic and nuclear extracts were prepared from Candid#1 JUNV-infected A549 cells at different h p.i., followed by measuring IRF3 protein levels in each subcellular fraction by SID-SRM. This analysis revealed that a low level of IRF3 nuclear translocation could be first detected at 6 h p.i. that peaked at 24 h p.i. and was maintained elevated at 48 h p.i. (Figure 1C). The percentage of total IRF3 translocated into the nucleus was 2.4% and 4.8% at 6 and 24 h p.i., respectively. In comparison, only 0.71% and 1.5% of IRF3 was detected in nuclear fractions of mock-infected cells at 6 and 24 h p.i., respectively. In a control experiment, IRF3 nuclear translocation was readily detected in cells transfected with poly (IC) (Figure S1). These results indicated that moderate levels of nuclear translocation of endogenous IRF3 were induced during early stage of Candid#1 JUNV infection, which likely contributed to type I IFN production and signaling (Figure 1A).

### IFN production during Candid#1 JUNV infection

**Figure 2:**
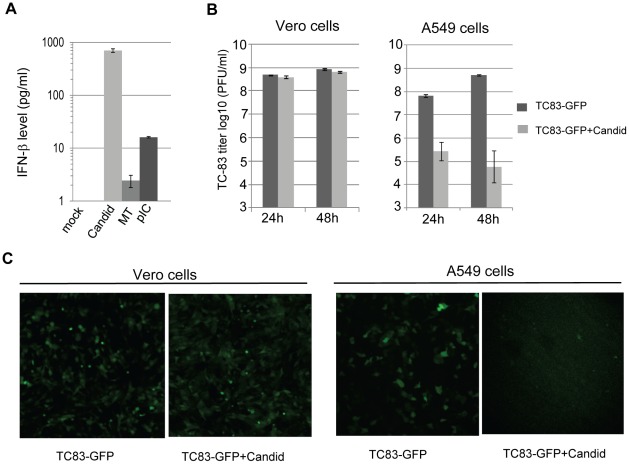
IFN induction in Candid#1 JUNV-infected cells. (A) A549 cells were mock-infected (mock) or Candid#1 JUNV infected (Candid) (MOI of 1) for 24 h. Levels of secreted IFN-β protein in supernatants were measured by IFN-β ELISA. As a control, cells were mock transfected (MT) or transfected with poly (IC) (pIC) for 24 h. (B) A549 cells and Vero cells were mock-infected or infected with Candid#1 JUNV (MOI of 1) for 24 h. Then cells were super-infected with VEEV TC83-GFP (MOI of 0.1). At 24 h and 48 h p.i., tissue culture supernatants were harvested and virus titers were determined by plaque assay in BHK cells. (C) Vero and A549 cells were mock-infected or infected with Candid#1 JUNV for 24 h followed by super-infection with VEEV TC83-GFP for 24 h. VEEV TC83-driven GFP expression in infected cells was assessed by fluorescence microscopy. TC83-GFP: cells mock-infected with Candid#1 JUNV and then infected with VEEV TC83-GFP virus. TC83-GFP+Candid: cells infected with Candid#1 JUNV and then super-infected with VEEV TC83-GFP virus.

We performed ELISA to directly measure the production of IFN-β protein in tissue culture supernatant (TCS) from Candid#1 JUNV infected A549 cells. At 24 h p.i., Candid#1 JUNV infection induced 701 pg/ml of IFN-β production in TCS, while poly (IC) transfection by Lipofectamine 2000 resulted in a lower level (16 pg/ml) of IFN production (Figure 2A). To identify the biological potency of type I IFN production in Candid#1 JUNV infected cells, we examined whether these A549 cells were resistant to super-infection with the IFN sensitive VEEV TC83-GFP virus [41]. As a control, we characterized the response of Candid#1 JUNV-infected Vero cells (IFN-deficient) to super-infection with VEEV TC83-GFP. VEEV-TC83 virus titers were determined by plaque assay in BHK cells as reported [42]. VEEV-TC83 grew to similar titers in both mock- and Candid#1 JUNV-infected Vero cells (Figure 2B, left panel). In contrast, VEEV-TC83 virus titers were reduced by more than 2.5- and 3.5-log in Candid#1 JUNV-infected A549 cells at 24 and 48 h p.i., respectively (Figure 2B, right panel). This result suggested that A549 cells became resistant to other RNA virus infection after Candid#1 JUNV infection.

We also monitored VEEV virus replication in mock- and Candid#1JUNV-infected Vero and A549 cells by assessing the VEEV TC83 virus-driven GFP expression level. Consistent with the results of VEEV TC83-GFP infectious progeny production, replication of VEEV was severely affected in Candid#1 JUNV-infected A549, but not in Vero cells (Figure 2C). In Vero cells, similar numbers of GFP positive cells were identified in mock-infected sample (205 counts) and Candid#1 JUNV-infected sample (190 counts) (Figure 2C). Whereas in A549 cells, the numbers of GFP positive cells were remarkably different between mock-infected sample (75 counts) and Candid#1 JUNV-infected sample (2 counts).

### Effect of infection with the pathogenic Romero strain of JUNV on activation of the IFN pathway

**Figure 3:**
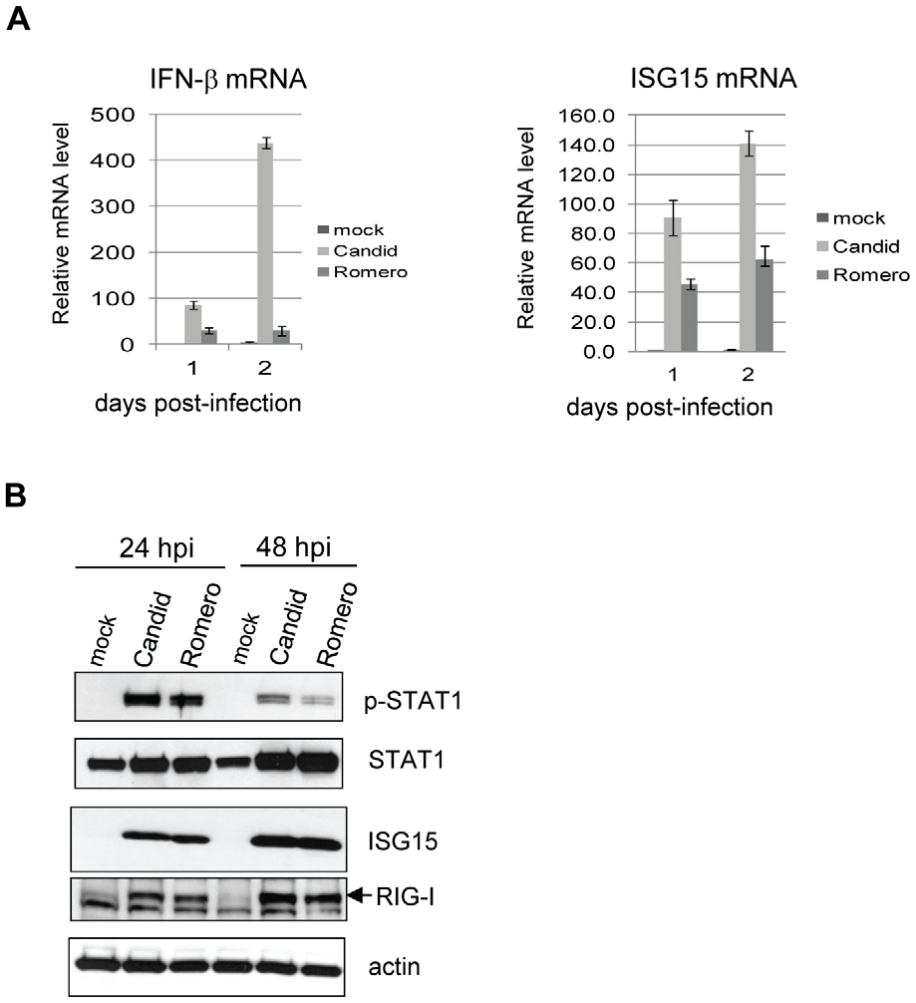
Activation of IFN pathway by both vaccine and pathogenic strains of JUNV. A549 cells were mock-infected or infected with Candid#1 or Romero strains of JUNV (MOI of 1) for the indicated times. (A) Total RNAs were extracted. IFN-β and ISG15 mRNA levels were determined by real-time qRT-PCR and normalized to GAPDH mRNA levels and presented as relative levels to these of mock-infected sample at day 1. Data shown are the mean of three experiments (error bar, S.D). (B) Protein lysates were prepared and subjected to western blotting analysis to detect phosphorylated STAT1 (p-STAT1), STAT1 (STAT1), ISG15 (ISG15), RIG-I (RIG-I) and β-actin (actin) proteins.

In an effort to understand the mechanisms underlying the attenuation of Candid#1 vaccine strain of JUNV, we compared the effect of virus infection on activation of type I IFN pathway between the non-pathogenic (Candid#1) and pathogenic (Romero) strains of JUNV in A549 cells. These studies were performed at the BSL-4 facilities at UTMB. Both Candid#1 and Romero virus strains induced noticeable levels of IFN-β and ISG15 mRNA expression in A549 cells as determined by real-time qRT-PCR (Figure 3A). IFN-β mRNA expression increased by 31-fold and 29-fold in Romero JUNV infected cells at days 1 and 2 p.i., respectively, meanwhile ISG15 mRNA was upregulated by 45-fold and 62-fold in the same samples. Candid#1 JUNV-infected cells expressed higher levels of IFN-β and ISG15 mRNAs than Romero JUNV-infected cells (Figure 3A). We also performed Western blotting analysis to determine the protein levels of ISGs (Figure 3B). Both Candid#1 and Romero strains of JUNV induced ISG15 protein expression at similar levels, along with similarly elevated levels of STAT1, including its phosphorylated form, and RIG-I protein. However, STAT1 phosphorylation was stronger in cells infected by Candid#1 JUNV than by Romero JUNV at 48 h p.i., implying the possibility that IFN signaling may be more robust in cells infected with the attenuated vaccine strain Candid#1 JUNV.

### Contribution of RIG-I to activation of the type I IFN pathway in Candid#1 JUNV-infected cells

**Figure 4:**
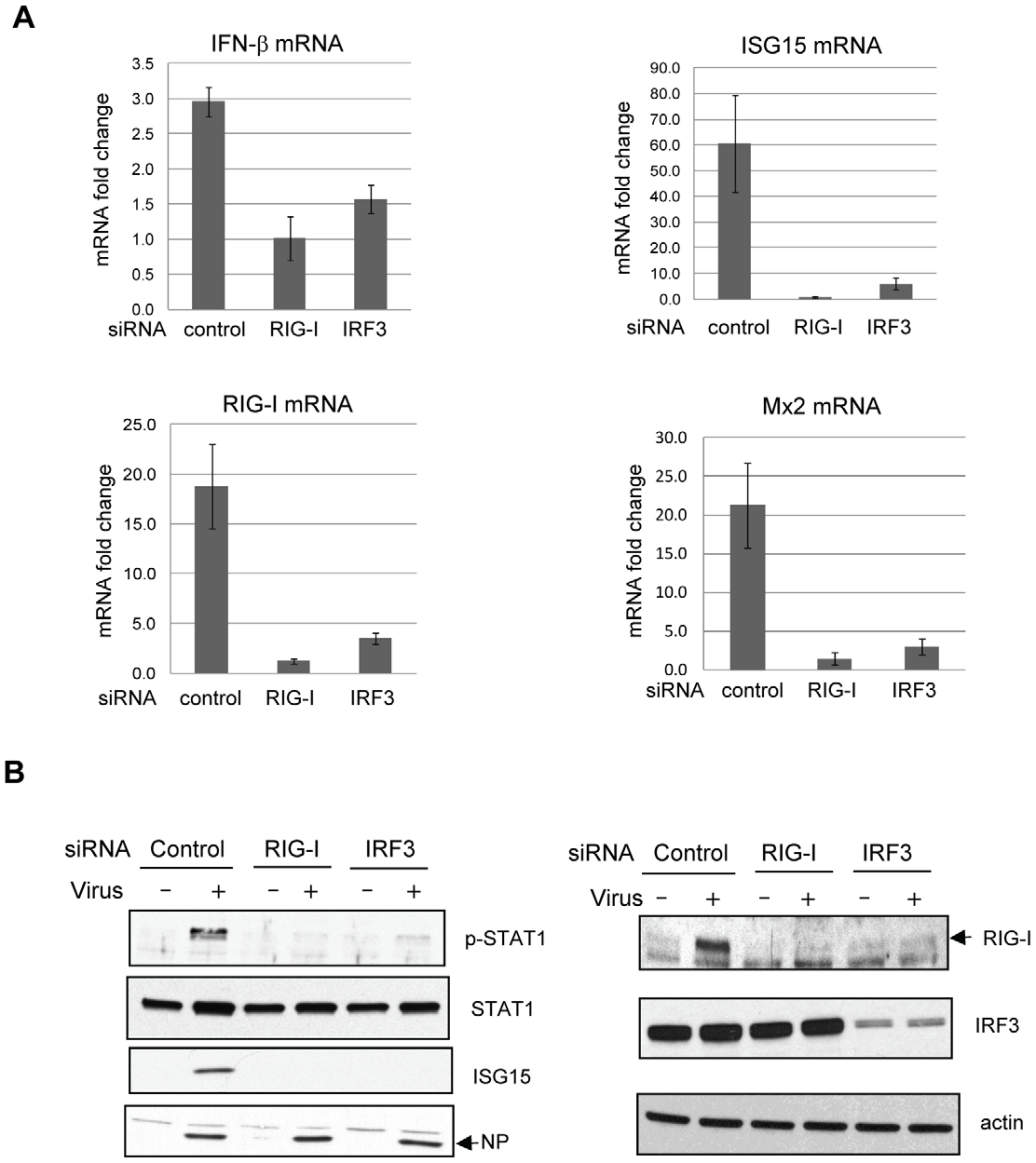
RIG-I is the sensor for IFN pathway activation. A549 cells were transfected with siRNAs targeting RIG-I or IRF3, as well as with a nontargeting control siRNA. At 48 h post-transfection, cells were mock-infected or infected with Candid#1 JUNV at an MOI of 1. (A) Total RNAs were extracted and levels of IFN-β, ISG 15, RIG-I and Mx2 mRNA were determined by RT-qPCR. Data were normalized to the levels of GAPDH mRNA and shown as fold-induction relative to mock-infected cells transfected with the corresponding siRNA. Values are the mean of three experiments (error bar, S.D). (B). In parallel, protein lysates were prepared from cells. Western blotting assays were performed to determine the protein levels of phosphorylated STAT1 (p-STAT1), STAT1 (STAT1), ISG15 (ISG15), viral NP protein (NP), RIG-I (RIG-I), IRF3 (IRF3) and β-actin (actin).

The cytoplasmic PRR RIG-I is involved in sensing many RNA viruses and triggering the host IFN response that involves the participation of the adaptor MAVS and downstream IRF3. Subsequently, activated IRF3, in cooperation with NF-κB and AP1 transcriptional factors, induces type I IFN production [17], [22]. Accordingly, we investigated whether the RIG-I/IRF3 pathway was responsible for sensing JUNV infection and consequently for induction of type I IFN. For this purpose, we studied the effect of siRNA-mediated knock-down of RIG-I and IRF3 expression on Candid#1JUNV-induced activation of type I IFN in A549 cells. A549 cells were transfected with siRNA for RIG-I, IRF3, or non-targeting control siRNA. After 48 h, cells were infected with Candid#1 JUNV for 24 h followed by analysis of RNA and protein samples with qRT-PCR and Western Blotting. The efficacy of gene knock-down at protein level was confirmed by Western blotting (Figure 4B). The levels of Candid#1 JUNV-induced IFN-β, ISG15, RIG-I and Mx2 mRNA expression were substantially impaired in A549 cells transfected with siRNA for RIG-I or IRF3 (Figure 4A). This result was further validated by Western blotting assay. In cells transfected with control siRNA, Candid#1 JUNV infection led to induction of ISG15 protein expression and STAT1 phosphorylation as expected (Figure 4B). Similarly, the level of RIG-I protein, an ISG product, also increased. In contrast, ISG 15 protein expression was undetectable during Candid#1 JUNV infection in cells transfected with siRNAs to RIG-I or IRF3. In IRF3 siRNA transfected cells, the level of RIG-I protein remained comparable to the basal level of RIG-I in control siRNA-transfected, mock-infected cells. RIG-I or IRF3 knock-down also led to diminished STAT1 phosphorylation in virus-infected cells. These data indicated that RIG-I/IRF3 pathway was required for activation of type I IFN pathway in response to Candid#1 JUNV infection.

### Sensitivity of non-pathogenic Candid#1 and pathogenic Romero strains of JUNV to IFN treatment

**Figure 5:**
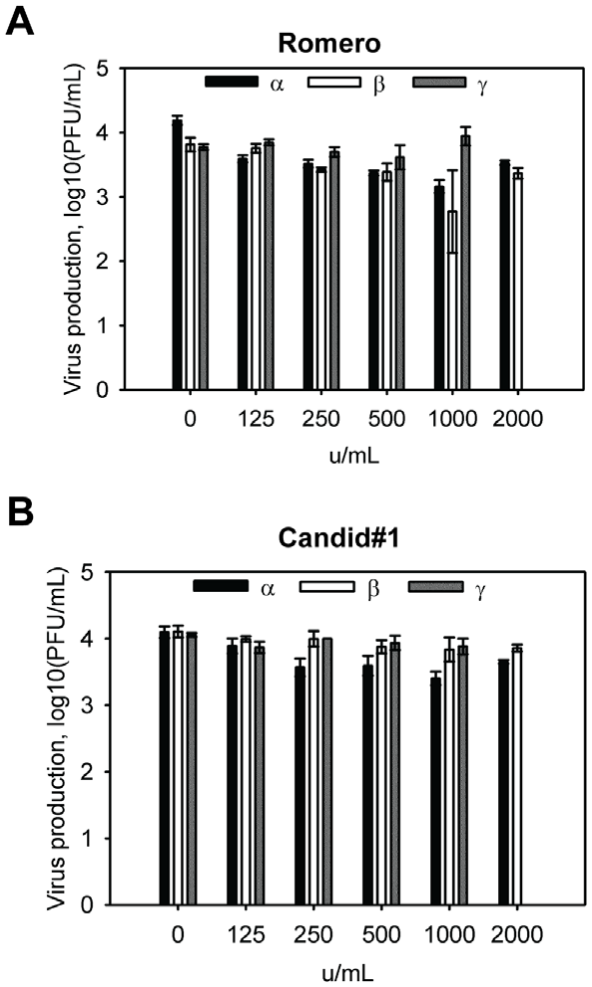
Sensitivity of JUNV to IFN pretreatment. Vero cells were treated with IFN-α, β or γ at the indicated concentrations for 8 h and then infected with Romero JUNV (A) or Candid#1 JUNV (B) at an MOI of 0.1. At 2 days p.i., supernatants were collected and assayed for virus production by plaque assay. Due to the severe cytotoxicity caused by IFN-γ treatment at 2000 U/ml, virus infection was not performed at this condition. Data represent the average of three replicates ±SEM.

Our observation that both non-pathogenic Candid#1 and pathogenic Romero strains of JUNV activated the type I IFN pathway raised the question of whether IFN could substantially inhibit the growth of these viruses. Past studies showed the insensitivity of some arenaviruses to IFNs [43]–[46]. We therefore characterized the IFN sensitivity of the Candid#1 and Romero strains of viruses. To this purpose, we treated Vero cells with IFN-α, β or γ at various concentrations for 8 h prior to virus infection and determined virus production at 48 h p.i.. Both Candid#1 and Romero strains were resistant to IFN-γ treatment in all conditions tested (Figure 5). Pre-treatment with IFN-β or IFN-α at a high concentration (1000 IU/ml) led to only minimal reduction in virus titer (less than 1-log) for both Candid#1 and Romero strains of viruses. In control experiments, IFN-β treatment at lower concentration (125 IU/ml) was sufficient to inhibit VSV virus production by more than 4-log (Figure S2). We observed similar results in Vero cells pre-treated with IFNs for 24 h prior to virus infection (data not shown). These data indicated that both Candid#1 and Romero strains of JUNV are surprisingly resistant to the antiviral effects of IFNs.

## Discussion

The central finding we report in this study is that infection of human A549 cells with either non-pathogenic Candid#1 or pathogenic Romero strains of JUNV resulted in activation of the type I IFN pathway, but both strains of JUNV were resistant to the antiviral effects of type I IFN. Consistently, it has been found that a pathogenic strain of JUNV induced IFN-β mRNA expression in human hematopoietic progenitor CD34^+^ megakaryocytes [47]. Our finding is further in agreement with clinical reports showing elevated levels of cytokines, including IFN-α, TNF-α, IL-6 and IL-10, in AHF patients [14]–[16], [26]–[30]. Clinical data also suggest that high levels of IFN-α in AHF patient might be correlated to poor prognosis [14], [27], yet the role of IFN and cytokine production in AHF pathogenesis remains to be elucidated. Interestingly, infection by LASV often triggers only weak immune responses and is associated with minimal noticeable pathology [1], [12], which usually does not correlate with the disease outcome, suggesting the pathogenesis of these two HF arenaviruses, JUNV and LASV, are quite distinct.

TLR2 has been reported to act as PRR for Candid#1 GP in mouse macrophages [32], but the role of TLR2 as PRR for JUNV in human cells remains to be determined. TLR2 or TLR4 are not expressed at detectable levels in A549 cells [48], suggesting that different PRR sensors might be utilized in JUNV recognition in different cell types. Accordingly, our data revealed that RIG-I was an important host sensor of JUNV infection in A549 cells. MDA5, another main cytoplasmic PRR, has been found crucial in host recognition of the prototypic arenavirus LCMV [49]. Further studies would be required to clarify the role of MDA5 in JUNV infection.

Infection with the pathogenic Romero strain of JUNV has been reported not to induce measurable levels of IFN-β, IFN-α, TNF-α, IL-10 or IL12, in primary human monocytes and macrophages [31]. This observation is in contrast to data reported here and in clinical reports. It is possible that this discrepancy is due to cell type differences or the relatively low levels of virus growth in human macrophages [31]. Interestingly, in agreement with our findings in A549 and Vero cells, cytokines had no direct inhibitory effect on Romero JUNV growth in macrophages [31].

The activation of the RIG-I/IRF3 pathway in JUNV-infected cells is supported collectively by transcriptional changes, type I IFN production, ISG expression, IRF3 nuclear translocation and STAT-1 phosphorylation in this study. These findings initially appear to be in conflict with previous observations documenting that NPs of many arenaviruses, including NP derived from JUNV, are capable of interfering with type I IFN induction [33]–[35], [49] through interaction with RIG-I and MDA5 [49] and/or inhibition of the nuclear translocation and transcriptional activity of IRF3 [33]–[35]. In addition, the Z protein of New World arenaviruses, has been reported to interact with RIG-I and inhibit its function [50]. It has been also proposed that arenaviruses are able to evade RIG-I recognition by virtue of the 5′ppp-nucleotide overhang at the double stranded RNA structures formed via terminal sequence complementarity of arenavirus genomic and antigenomic RNAs [51], [52]. However, these studies, mostly based on plasmid-based over-expression systems or in vitro biochemical analyses, do not directly study the IFN response in JUNV–infected cells. It is worth noting that, in cell-based reporter assay systems, over-expression of JUNV NP indeed was not able to completely abolish the activation of the RIG-I/IRF3 pathway in response to subsequent Sendai virus infection [34], which implies the occurrence of leaky activation of RIG-I/IRF3 pathway in these studies. Therefore, it is plausible that during the early stages of JUNV infection, low levels of NP protein might not be sufficient to completely circumvent the activation of the RIG-I/IRF3 pathway and induction of type I IFN response. Increased levels of NP at later times during infection may efficiently inhibit the RIG-I/IRF3 pathway, thus enabling virus to down-regulate the type I IFN response.

Our results demonstrate that both the non-pathogenic Candid#1 and pathogenic Romero strains of JUNV are capable of activating the type I IFN pathway in human A549 cells. However, higher levels of IFN-β and ISG15 mRNAs (Figure 3A), as well as increased levels of STAT1 phosphorylation (Figure 3B) were identified in Candid#1 JUNV-infected cell than in Romero JUNV-infected cells. These findings would suggest that the attenuated Candid#1 strain might be recognized more efficiently by RIG-I/IRF3 than Romero strain, or that Romero strain may be more effective in counteracting the induction of type I IFN. It is possible that sequence variation between two virus strains might determine these subtly different host responses. Future studies are required to identify whether the difference in type I IFN pathway response between non-pathogenic and pathogenic strains of JUNV has any implication in viral pathogenicity and attenuation. Notably, both Candid#1 and Romero strains of JUNV were only modestly sensitive to the antiviral effects of IFN pretreatment, yet Candid#1 JUNV-infected A549 cells still established an efficient antiviral state against another IFN-sensitive RNA virus. In IFN sensitivity assay, it is possible that the effect of IFN treatment might decline over the time. The slowly growing JUNV might benefit from abated IFN effect and eventually replicate more efficiently later. Our data are in agreement with previous studies showing the resistance of some arenaviruses to IFNs [43], [44], [46]. Although the pathogenicity of many viruses may be associated with their sensitivity to IFN [53], [54], attenuation of Candid#1 vaccine strain is unlikely due to enhanced sensitivity to IFN.

In conclusion, we have shown that both the non-pathogenic Candid#1 and pathogenic Romero strains of JUNV induced a RIG-I dependent type I IFN response in infected cells, but both Candid#1 and Romero strains of viruses were insensitive to the antiviral effects of IFN despite the fact that Candid#1 JUNV-infected cells mounted an effective antiviral state against another IFN-sensitive RNA virus. Future studies are warranted to investigate the importance of type I IFN signaling pathway in AHF pathogenesis in animal models. Gaining insights into JUNV pathogenesis will potentially enable us to design antiviral therapeutics to modulate the host antiviral response, limit infection and improve the disease outcome.

## Supporting Information

Figure S1 **IRF3 nuclear translocation in poly(IC)-transfected A549 cells.** A549 cells were transfected with poly (IC) by electroporation. At 0 hr and 1 hr after treatment, cytoplasmic extract (Cyto) and nuclear extract (Nu) were prepared to measure the IRF3 protein level in cytoplasm and nuclear fractions by SID-SRM analysis.
(TIF)

Figure S2 **Sensitivity of VSV to IFN pretreatment.** Vero cells were treated with IFN-α, β or γ at the indicated concentrations for 8 h and then infected with VSV at an MOI of 0.1. At 12 h p.i., supernatants were collected and assayed for virus production by plaque assay. Data represent the average of three replicates ±SEM.
(TIF)

Text S1 **Methods for quantitative proteomic analysis (LC-SRM-MS analysis).**
(DOC)

Table S1 **SRM parameters of SRM assays of IRF3.** Masses listed are for the natural forms of the peptides.
(DOC)
