# Supplementary figures and images for: Junín Virus Infection Activates the Type I Interferon Pathway in a RIG-I-Dependent Manner
Source: PLoS Negl Trop Dis. 2012 May 22;6(5):e1659. doi: 10.1371/journal.pntd.0001659 (PMC3358329; doi:10.1371/journal.pntd.0001659)

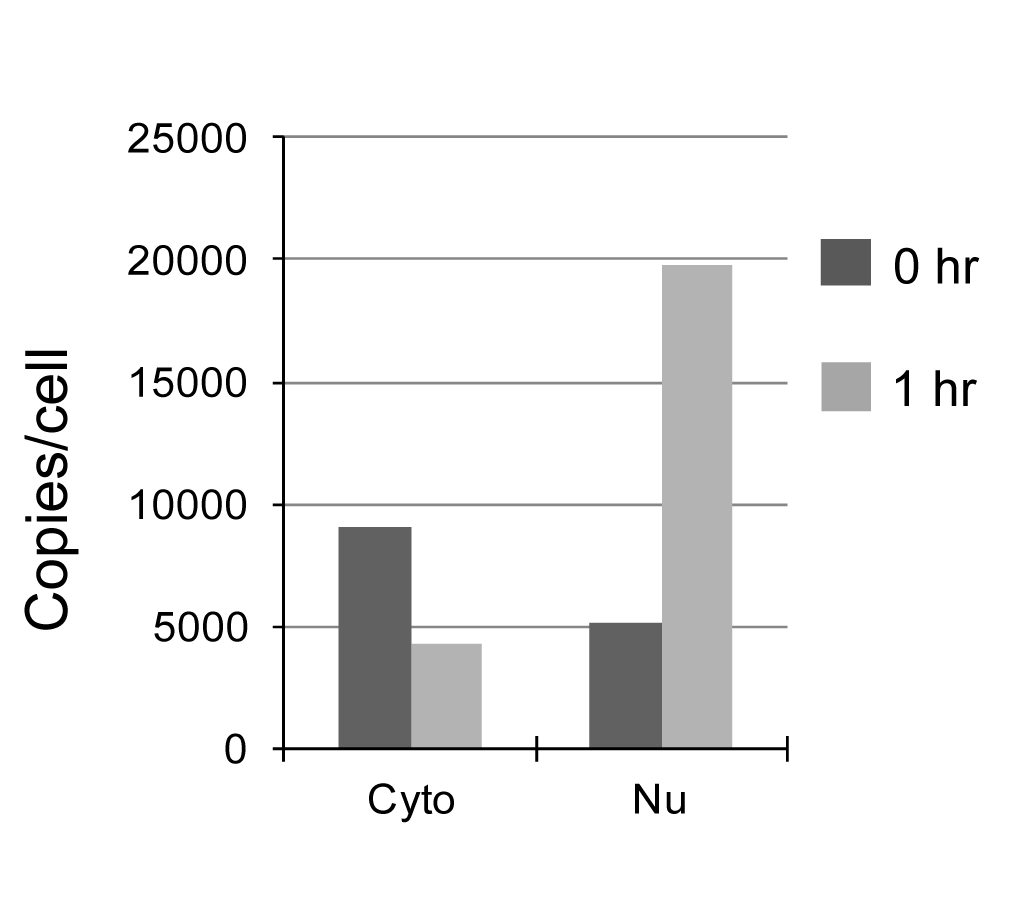

Supplement: Figure S1 — IRF3 nuclear translocation in poly(IC)-transfected A549 cells. A549 cells were transfected with poly (IC) by electroporation. At 0 hr and 1 hr after treatment, cytoplasmic extract (Cyto) and nuclear extract (Nu) were prepared to measure the IRF3 protein level in cytoplasm and nuclear fractions by SID-SRM analysis. (TIF) [file pntd.0001659.s001.tif]

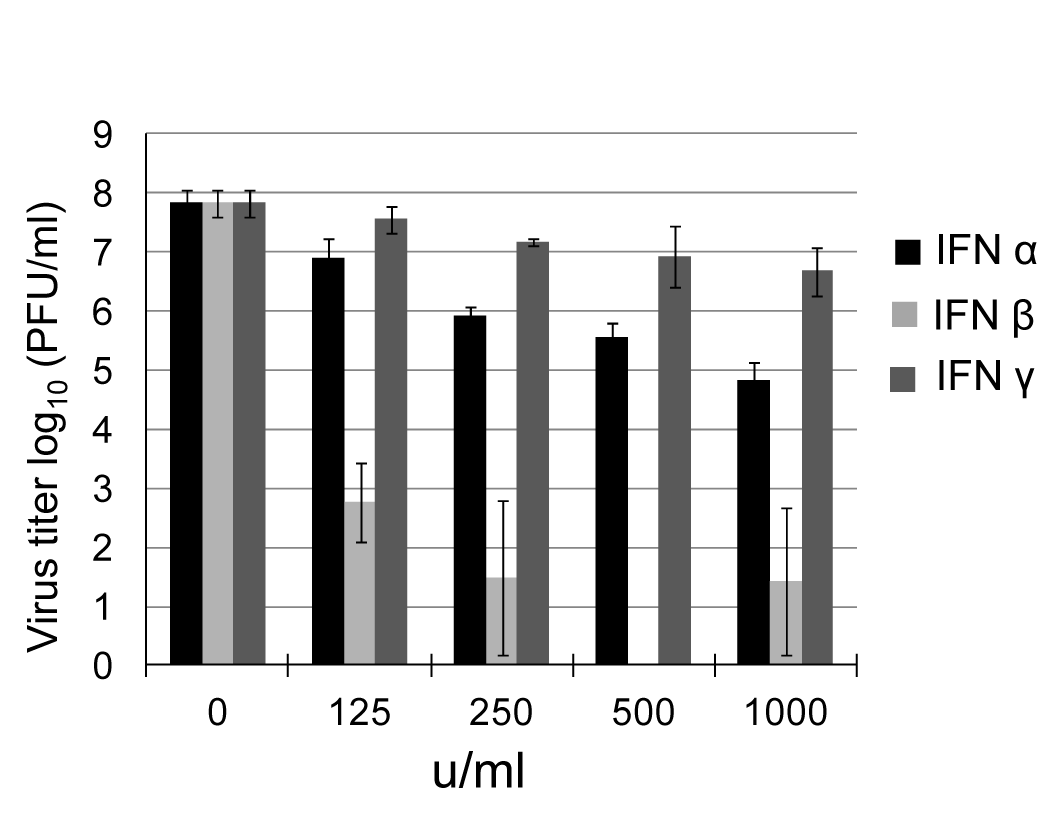

Supplement: Figure S2 — Sensitivity of VSV to IFN pretreatment. Vero cells were treated with IFN-α, β or γ at the indicated concentrations for 8 h and then infected with VSV at an MOI of 0.1. At 12 h p.i., supernatants were collected and assayed for virus production by plaque assay. Data represent the average of three replicates ±SEM. (TIF) [file pntd.0001659.s002.tif]
