# Supplementary material for: Junín Virus Infection Activates the Type I Interferon Pathway in a RIG-I-Dependent Manner
Source: PLoS Negl Trop Dis. 2012 May 22;6(5):e1659. doi: 10.1371/journal.pntd.0001659 (PMC3358329; doi:10.1371/journal.pntd.0001659)
Supplement: Text S1 — Methods for quantitative proteomic analysis (LC-SRM-MS analysis). (DOC) [file pntd.0001659.s003.doc]

**Text S1 Methods for quantitative proteomic analysis (LC-SRM-MS analysis).**

**Trypsin digestion of protein extracts.** Protein extracts were denatured with 8 M guanidinium HCl and a 50 µg aliquot of each sample was reduced with 10 mM DTT for 30 min at RT. Cysteinyl residues were alkylated with 30 mM of iodoacetamide for 2 h at 37°C. The samples were diluted 10-fold with 100 mM ammonium bicarbonate, and digested with 2 μg of trypsin overnight at 37°C. The trypsin digestion was stopped by adding 10% TFA. The volume of each sample was adjusted with water to 400 µl. The tryptic digests were dried and resuspended in 5% formic acid-0.01% TFA prior to analysis.

Stable isotope standard (SIS) peptide stocks were diluted to a concentration of 10 fmol/ µl with 0.01% TFA. Before LC-SRM-MS analysis, 30 µl of each tryptic digest was mixed with 10 µl of each SIS peptide and desalted with a Waters Sep-Pak C18 cartridge (Milford, MA).

**Selected Reaction Monitoring (SRM) assays.** Signature peptides that stoichiometrically represented the protein candidate were selected based on standard criteria . The highest MS responding peptides for each protein were predicted by ESP Predictor . For each target protein, three to five peptides with best ESP prediction score were chemical synthesized in unpurified, isotope labeled format and their sensitivity and detectability were empirically determined by LC-SRM. The peptides with highest MS response were selected as the high-responding signature peptide for each target protein. These signature peptides are listed in Supplementary data Table S1.

For each high-responding signature peptides, 3-5 y-ions that exceeded precursor ion m/z were chosen. The selection of fragment ions were on the basis of MS/MS spectra of the peptide from SRM-triggered MS/MS experiments, the highest intensity fragment ions in MS/MS were selected to maximize the sensitivity of detection. The collision energy (CE) for each signature peptide was predicted by the generic formula CE = 0.034 x (precursor ion m/z) + 3.314. The selected Q1/Q3 transitions and their CE are tabulated in supplementary Table S1.

**Synthesis of Native and Stable (SIS).** Native tryptic peptides were synthesized using N-(9-fluorenyl) methoxycarbonyl chemistry. SIS peptides were commercially synthesized incorporating isotopically labeled [^13^C_6_^15^N_4_] Arginine or [^13^C_6_^15^N_4_] Lysine to a 98% isotopic enrichment (Sigma Aldrich). Both native and SIS peptides were HPLC purified to >98% purity. The molecular weights were measured with electrospray mass spectrometry and matrix-assisted laser desorption/ionization time-of-flight mass spectrometry. The specific peptide concentration was determined by amino acid analysis. Individual SIS peptide stocks (20 pmol/µl) were made in 80% ACN.

**LC-SRM-MS Analysis.**  LC-SRM-MS analysis was performed with a TSQ Vantage triple quadrupole mass spectrometer equipped with nanospray source (ThermoFinnigan, San Jose, CA). The online desalting and chromatography were performed using an Eksigent NanoLC-2D HPLC system (AB SCIEX, Dublin, CA). An aliquot of 10 μl of each of tryptic digests were injected on a C18 peptide trap (Agilent, Santa Clara, CA), desalted with 0.1 % formic acid at a flow rate of 2 μl/min for 45 min. Peptides were eluted from the trap and separated on a reverse-phase nano-HPLC column (PicoFrit™, 75 µm x 10 cm; tip ID 15 µm) packed in house using Zorbax SB-C18 (5-µm diameter particles, Agilent, Santa Clara, CA). Separations were performed using a flow rate of 500 nl/min with a 20-min linear gradient from 2-40% mobile phase B (0.1 % formic acid-90 % acetonitrile) in mobile phase A (0.1 % formic acid), followed by 0.1-min gradient from 40-90% mobile phase B and 5-min 90% mobile phase B. The TSQ Vantage was operated in high-resolution SRM mode with Q1 and Q3 set to 0.2 and 0.7-Da Full Width Half Maximum. All acquisition methods used the following parameters: 1800 V ion spray voltage, a 275 °C ion transferring tube temperature, a collision-activated dissociation pressure at 1.5 mTorr, and the S-lens voltage used the values in S-lens table generated during MS calibration. The MS raw files will be made accessible at <http://scmm.utmb.edu/faculty/zhao.htm> upon publication of the manuscript.

**SRM Data Analysis.** All SRM data were processed using Xcalibur® 2.1. The default values for noise percentage and base-line subtraction window were used. All data were manually inspected to ensure peak detection and accurate integration. The chromatographic retention time and the relative product ion intensities of the analyte peptides were compared to those of the SIS peptides. The variation of the retention time between the analyte peptides and their SIS counterparts should be within 0.05 min, and no significantly difference in the relative product ion intensities of the analyte peptides and SIS peptides were observed.
