# Supplementary material for: Junín Virus Infection Activates the Type I Interferon Pathway in a RIG-I-Dependent Manner
Source: PLoS Negl Trop Dis. 2012 May 22;6(5):e1659. doi: 10.1371/journal.pntd.0001659 (PMC3358329; doi:10.1371/journal.pntd.0001659)
Supplement: Table S1 — SRM parameters of SRM assays of IRF3. Masses listed are for the natural forms of the peptides. (DOC) [file pntd.0001659.s004.doc]

**Table S1.** SRM parameters of SRM assays of IRF3. Masses listed are for the natural forms of the peptides.

| **Gene Name** | **Swissprot No.** | **Sequence** | **Q1 m/z** | **Q3 m/z** | **Ion type** | **CE (V)** |
| --- | --- | --- | --- | --- | --- | --- |
| **IRF3** | Q14653 | **LVGSEVGDR** | 466.242 | 446.235 | y4 | 20 |
|  |  |  | 466.242 | 575.278 | y5 | 16 |
|  |  |  | 466.242 | 719.331 | y7 | 17 |
|  |  |  |  |  |  |  |
